# Supplementary material for: Robust trap effect in transition metal dichalcogenides for advanced multifunctional devices
Source: Nat Commun. 2019 Sep 12;10:4133. doi: 10.1038/s41467-019-12200-x (PMC6742650; doi:10.1038/s41467-019-12200-x)
Supplement: Supplementary file 1 — Supplementary Information [file 41467_2019_12200_MOESM1_ESM.pdf]

Supplementary Information

Robust trap effect in transition metal dichalcogenides for advanced multifunctional devices

Yin et al.

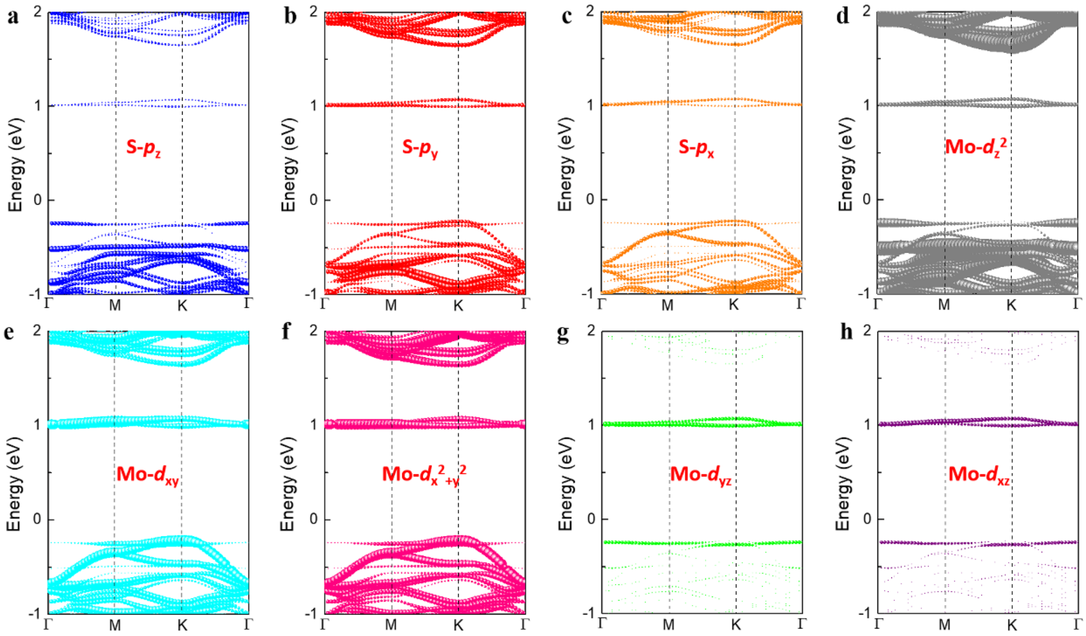

Supplementary Figure 1 Projected band structure of monolayer 2H-MoS_2 with a single SV at PBE level: S-p_z (**a**), S-p_y (**b**), S-p_x (**c**), Mo-d_{z^2} (**d**), Mo-d_{xy} (**e**), Mo-$d_{x^2+y^2}$ (**f**), Mo-d_{yz} (**g**) and Mo-d_{xz} (**h**). Fermi level is set to zero. This defective MoS_2 model was constructed from a (4 × 4) supercell. The larger the plotted point is, the stronger the corresponding electron states contribute to the defect levels. When the regulation of Se atoms on defect levels is considered, Mo-4*d* states are more likely to be affected compared with S-3*p* states. Hence, we mainly focus on the change in contribution of Mo-4*d* states to the defect levels after introducing Se atoms. Source data are provided as a Source Data file.

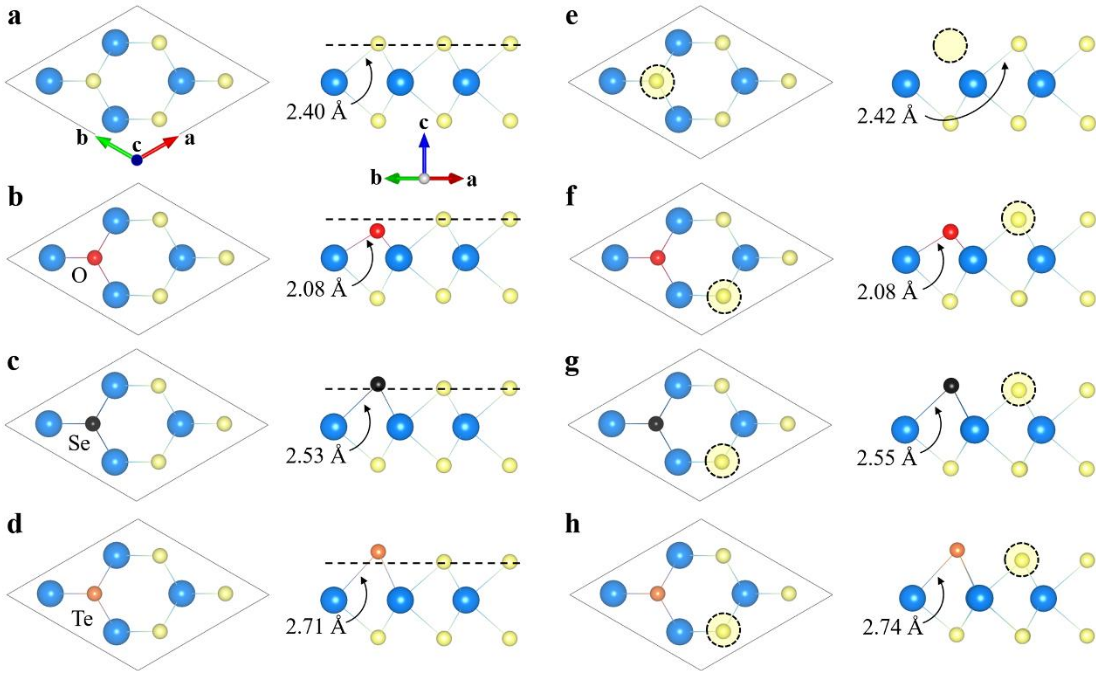

Supplementary Figure 2 Atomic structure of (2 × 2) supercell of monolayer MoS_2 and $MoS_{2x}X_{2(1-x)}$ (X = O, Se, Te) without (**a-d**) and with (**e-h**) one SV. Both top views (left) and side views (right) are shown. The bond length of Mo-X (X = S, O, Se or Te) is labeled on the bottom of side view. Mo, S, O, Se and Te atoms are represented by blue, yellow, red, black and orange balls respectively. The dotted line on the side view of atomic structure is used to show the structural change of $MoS_{2x}X_{2(1-x)}$ clearly. The SV is highlighted by pale yellow circle with black outline.

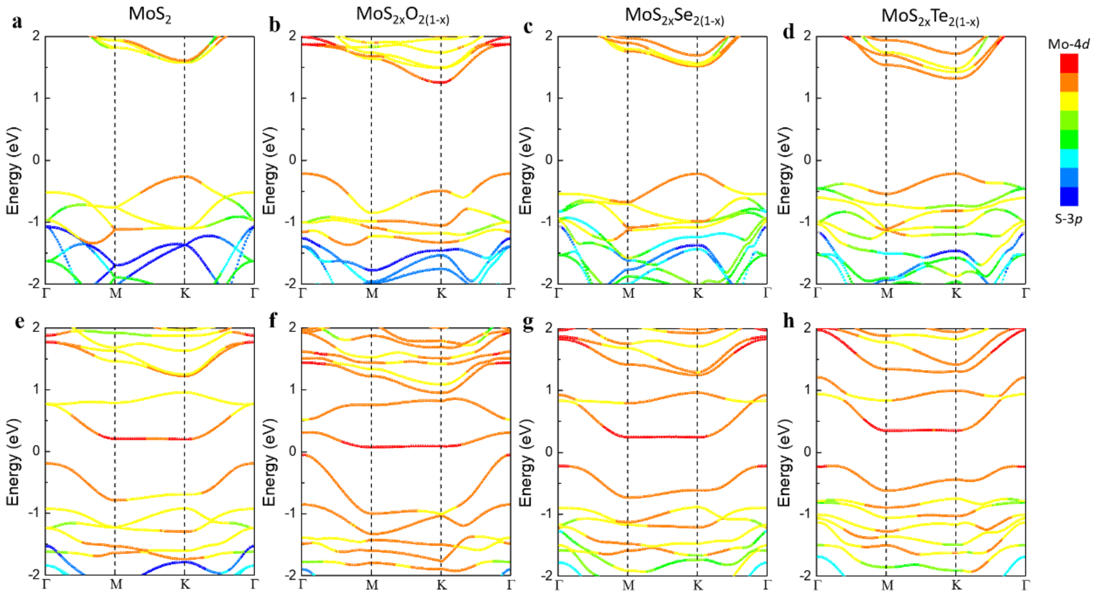

Supplementary Figure 3 Projected band structure of monolayer MoS_2 and $MoS_{2x}X_{2(1-x)}$ (X = O, Se, Te) without (**a-d**) and with (**e-h**) one SV. Fermi level is set to zero. The calculations were based on a (2 × 2) MoS_2 supercell. Compared with the moderate regulation of Se atom, the introduction of O or Te atom modifies the band structure of pristine MoS_2 more dramatically. Source data are provided as a Source Data file.

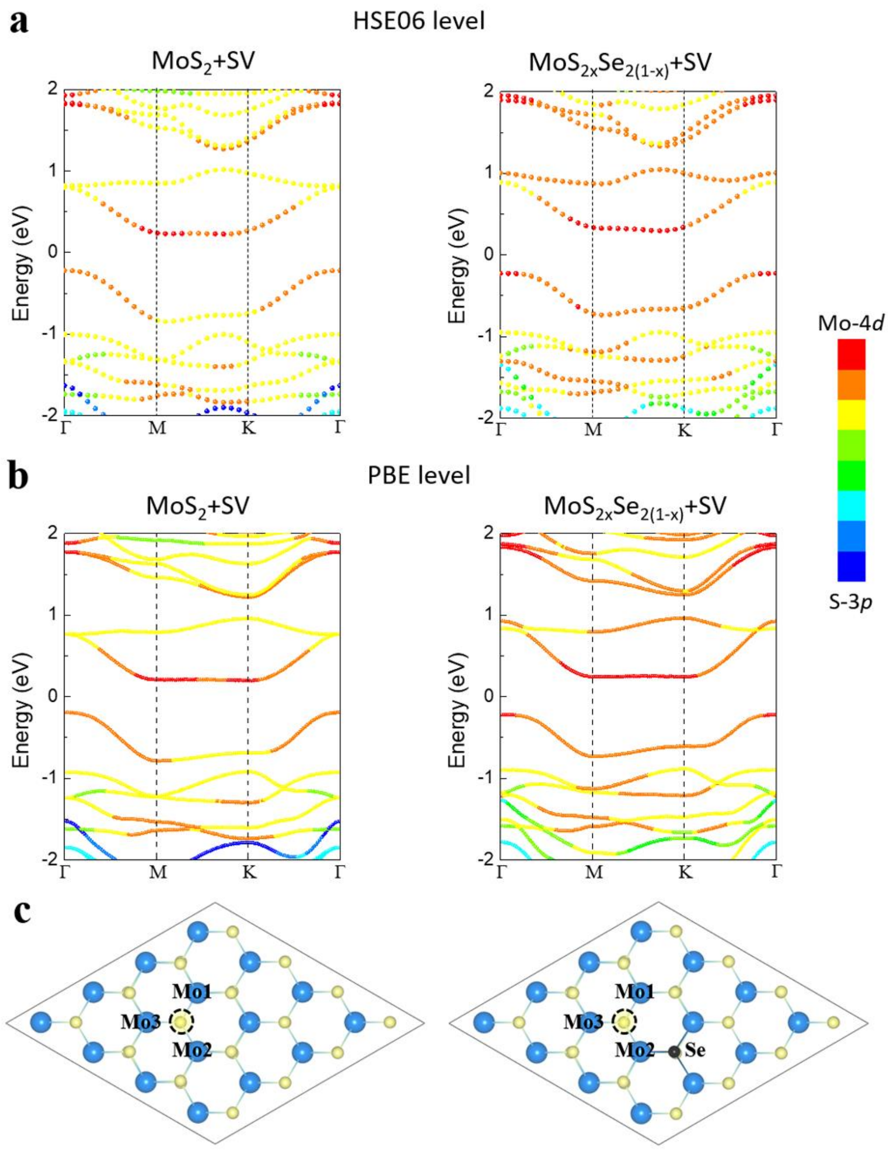

Mo location	PBE level		HSE06 level	
	MoS_2+SV	$MoS_{2x}Se_{2(1-x)}$+SV	MoS_2+SV	$MoS_{2x}Se_{2(1-x)}$+SV
Mo1	-0.65 |e|	+0.24 |e|	-1.27 |e|	-0.82 |e|
Mo2	-0.65 |e|	-0.19 |e|	-1.42 |e|	-1.25 |e|
Mo3	-0.68 |e|	+0.94 |e|	-0.80 |e|	-0.10 |e|

Supplementary Figure 4 Comparison between the calculation results obtained with HSE06 and PBE functional. (**a, b**) Band structures of (2 × 2) MoS_2 and $MoS_{2x}Se_{2(1-x)}$ monolayer with one SV at HSE06 level (**a**) and PBE level (**b**). (**c**) The Bader charge of (4 × 4) MoS_2 and $MoS_{2x}Se_{2(1-x)}$ monolayer with one SV at PBE and HSE06 level. The SV is highlighted by black dotted circle. Mo, S and Se atoms are represented by blue, yellow and black balls, respectively. The location of selected Mo atoms is marked on top panel and the corresponding Bader charge values are listed at bottom panel. The results obtained at PBE level shows the same trend as that of HSE06 level. Source data are provided as a Source Data file.

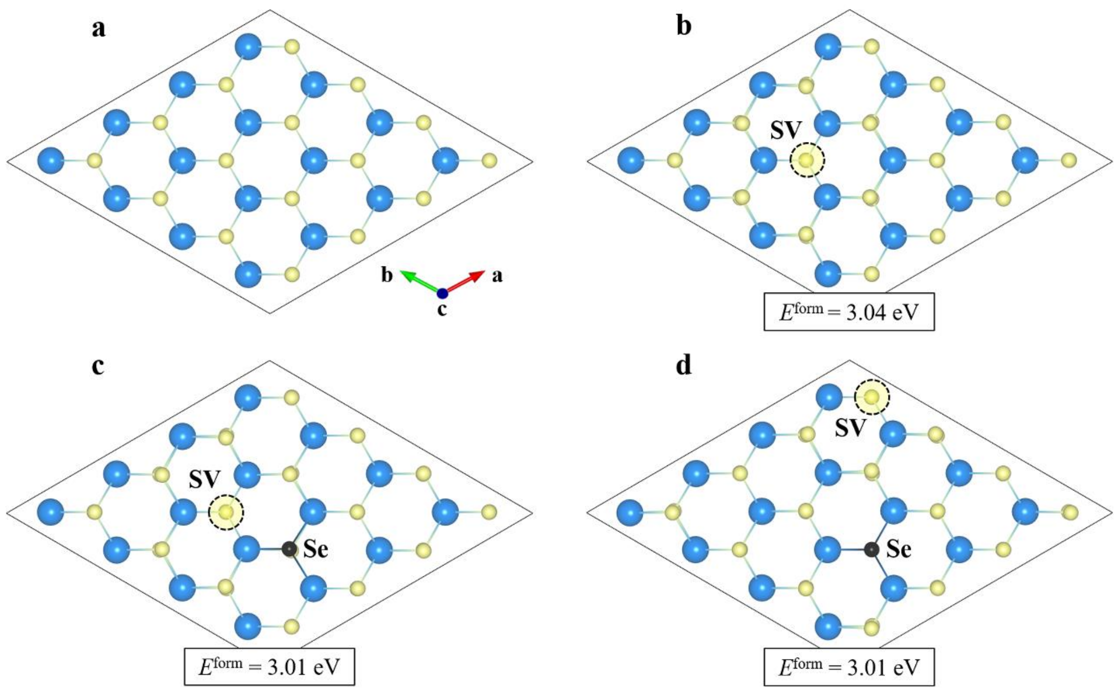

Supplementary Figure 5 (**a, b**) Atomic structure of (4 × 4) supercell of monolayer MoS_2 without (**a**) and with (**b**) one SV. (**c, d**) Atomic structure of (4 × 4) supercell of Se-doped MoS_2 with one SV, in which Se atom is close to (**c**) and far from (**d**) the SV. The SV is highlighted by pale yellow circle with black outline. Both the locations of SV and substituted Se atom are on the upper surface of MoS_2. Mo, S and Se atoms are represented by blue, yellow and black balls, respectively. The formation energy of one SV in pristine MoS_2 and Se-doped MoS_2 is labeled on the bottom of (**b**), (**c**) and (**d**), respectively. Their formation energies are 3.04, 3.01 and 3.01 eV, respectively. A positive SV formation energy means that the formation of SV is an endothermic reaction. The smaller the formation energy, the more easily it can form the SV. Therefore, SV is easier to form in Se-doped MoS_2, and its formation location is independent of the location of the substituted Se atom.

The formulas for calculating the formation energy of vacancy (E^{form}) and substitution by anions (E^{sub}) are as follows[1],

$E^{form} = E(\text{defect}) - E(\text{perfect}) + \mu_S$,

$E^{sub} = E(\text{Se+slab}) - E(\text{slab}) + \mu_S - \mu_{Se}$,

where E(defect) and E(perfect) represent respectively the total energy of a (4 × 4) supercell with and without one SV, E(Se+slab) and E(slab) represent respectively the total energy of a (4 × 4) supercell with and without one Se atom, μ_S and μ_{Se} are the chemical potential of a S and Se atom. The computed values of the μ_S and μ_{Se} chemical potentials are -3.464 and -3.309 eV/atom, obtained from bulk (space group: $R\bar{3}mH$) S and Se, respectively. Here, we focus on the E^{form} (E^{sub}) differences of an SV (substituted Se atom) at different sites, which is independent of the choice of μ_S (μ_{Se})[2].

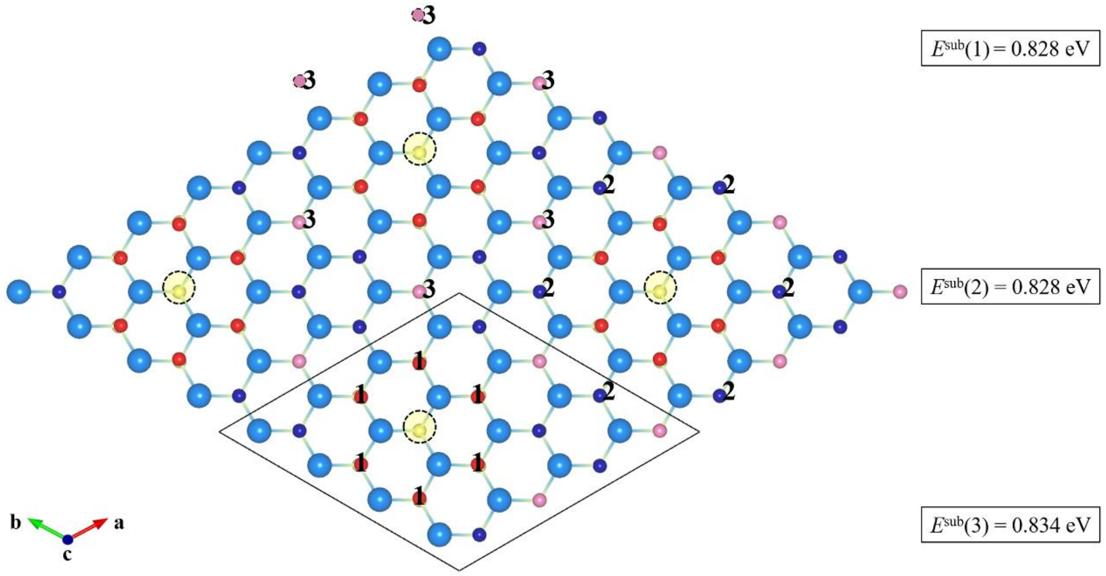

Supplementary Figure 6 Schematic diagram of all possible substitution locations of one Se atom in a (4 × 4) supercell of MoS_2 with one SV. Blue balls represent the cation sites. And other colorful (yellow, red, dark blue and pink) balls represent the anion sites. Location 1, location 2 and location 3 are shown by red, dark blue and pink balls, respectively. The SV is highlighted by pale yellow circle with black outline. From location 1 to location 3, the distance between the substituted Se atom and the SV increased gradually. The formation energy of substitution at location 1, 2 and 3 is labeled on the right. The formation energy of substitution of the nearest-neighbor site (location 1) and the next-nearest-neighbor site (location 2) is equal (0.828 eV), while the formation energy of substitution of location 3 is the largest (0.834 eV). It means that Se atom is more favorable to substitute S atom near SV. Therefore, when we model Se concentration (C_{Se}) from 3.13% (one Se atom) to 25.00% (eight Se atoms) in Se-doped MoS_2 with one SV, the S atoms at location 1 are prior to be replaced by Se atoms, then followed by location 2 and location 3.

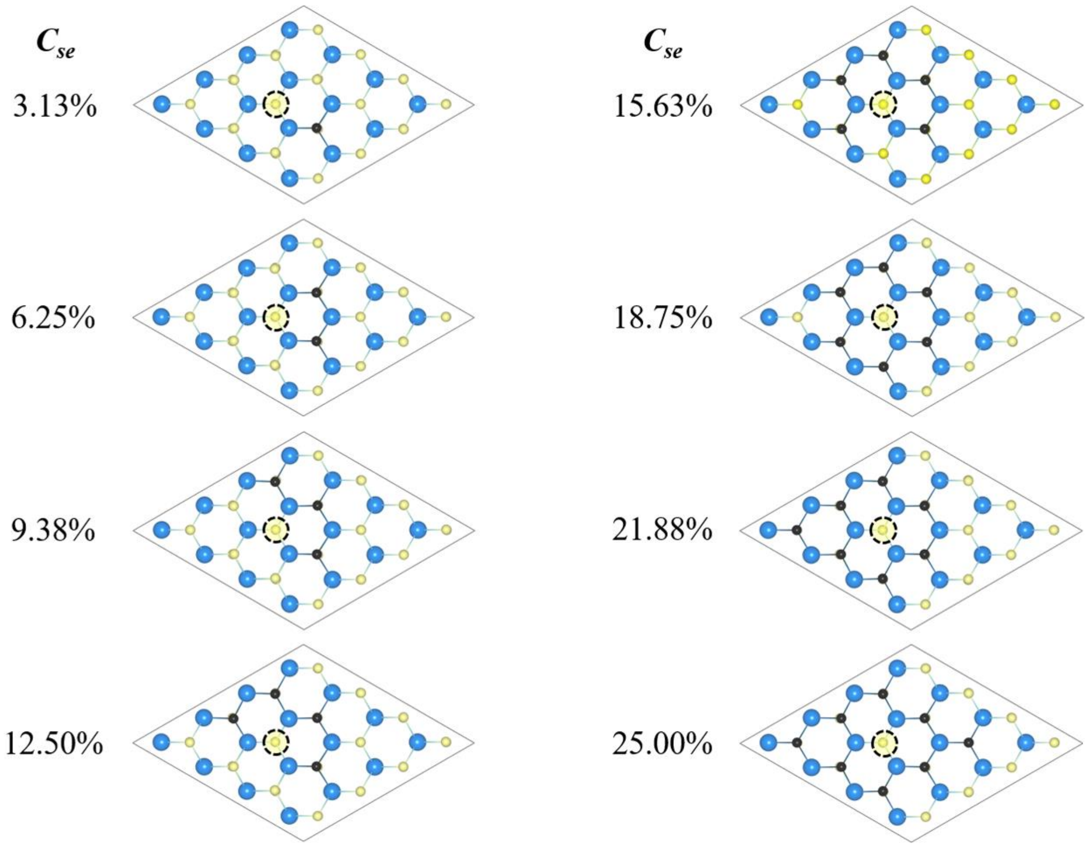

Supplementary Figure 7 Atomic structure of $MoS_{2x}Se_{2(1-x)}$ with increasing C_{Se} from 3.13% to 25.00%. Blue, yellow, and black balls represent the Mo, S, and Se atoms, respectively. The SV is highlighted by pale yellow circle with black outline.

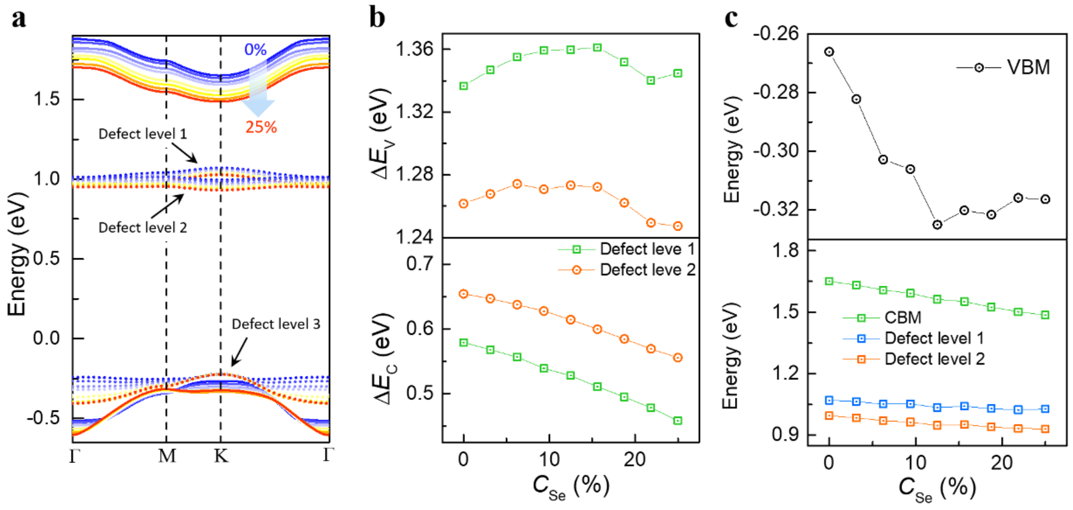

Supplementary Figure 8 Effects of the doped Se on the electronic structure of defective MoS_2. (**a**) The evolution of band structure of $MoS_{2x}Se_{2(1-x)}$ with increasing C_{Se} from 0% to 25%. Only the band edges (solid lines) and defect levels (dot lines) are shown. (**b**) The absolute energy positions of defect levels (DL-1 and DL-2), valence band maximum (VBM), and conduction band minimum (CBM) as a function of C_{se}. (**c**) The energy difference of VBM/CBM and defect levels (DL-1 and DL-2) as a function of C_{se}. The modulation of C_{Se} on VBM and CBM is more striking than that on defect levels. As the upper panel of Supplementary Fig. 3c shown, the change of energy different (ΔE_V) between VBM and DL-1 (DL-2) is an inverse parabola along with C_{Se}. And the maximal change values are only 24.6 meV (DL-1) and 12.3 meV (DL-2) relative to the case with 0% C_{Se}. For hole traps, the almost unchanged ΔE_V is large enough to avert the effects of thermal perturbation. On the other hand, the ΔE_C between CBM and DL-1 (DL-2) is monotonously decreased in the C_{Se} range of 0% to 25% (the bottom panel of Supplementary Fig. 3c). The reduction is not conducive to hole trapping. Therefore, introducing a right amount of Se atoms can bring about the potential of hole storage. Source data are provided as a Source Data file.

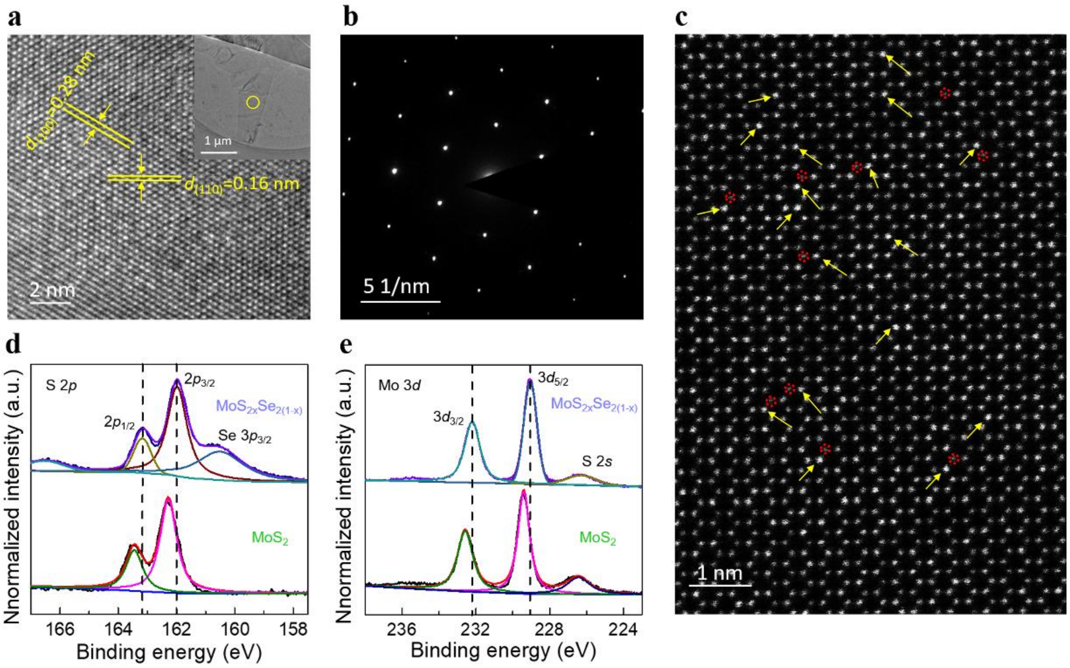

Supplementary Figure 9 Chemical and structural characterization. The high-resolution transmission electron microscopy (TEM) image (**a**), selected-area electron diffraction (SAED) pattern (**b**), and high-angle annular dark-field scanning transmission electron microscopy (HAADF-STEM) image (**c**) of an ultrathin nanosheet shown in the inset of (**a**). The extracted lattice distances of 0.28 nm and 0.16 nm are coincident with (100) and (110) planes of $MoS_{2x}Se_{2(1-x)}$ nanosheets[3], respectively. The corresponding SAED pattern with hexagonal symmetry indicates the high crystallinity. Moreover, the HAADF-STEM image shows distinct hexagonal arrangement of atoms with clear contrast, which further confirms the substitution of S atoms with Se atoms and the existence of S vacancies. The yellow arrows and the dashed red circles in (**c**) denote the possible sites of Se atoms and vacancies, respectively. (**d, e**) X-ray photoelectron spectroscopy (XPS) analysis of $MoS_{2x}Se_{2(1-x)}$ and MoS_2 in S 2*p*, Se 3*p*, and Mo 3*d* regions, respectively. In XPS spectra, the binding energy peak of Se $3p_{3/2}$ was detected only in the $MoS_{2x}Se_{2(1-x)}$ samples. In addition, there are obvious red shift in the core-level peaks of Mo 3*d* and S 2*p* in the $MoS_{2x}Se_{2(1-x)}$ samples compared to those of the undoped MoS_2, which manifests the presence of Se atoms and S vacancies[4,5]. Source data are provided as a Source Data file.

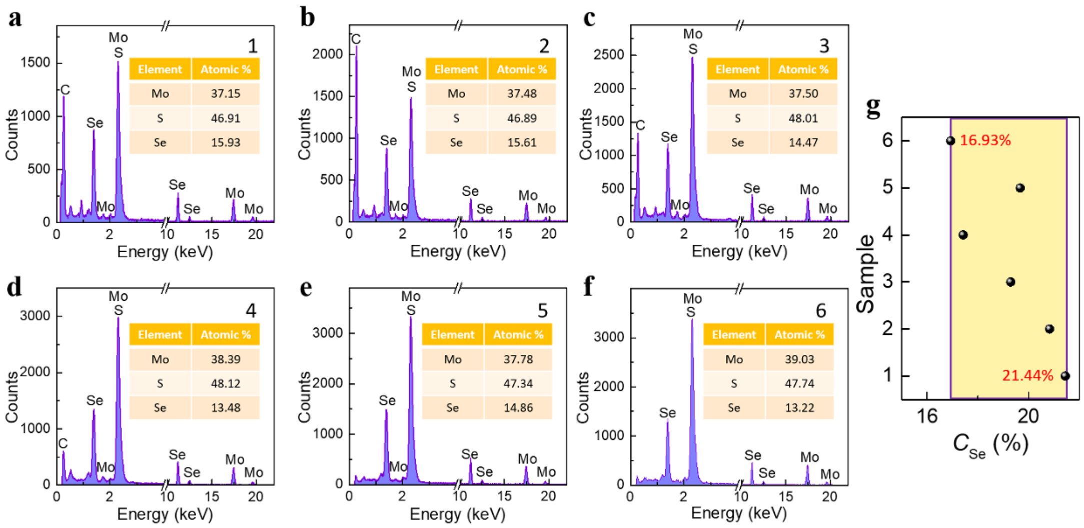

Supplementary Figure 10 Component analysis. (**a-f**) STEM-EDX spectrum of multiple $MoS_{2x}Se_{2(1-x)}$ nanosheets. (**g**) The summary of Se concentration of the $MoS_{2x}Se_{2(1-x)}$ crystal. Source data are provided as a Source Data file.

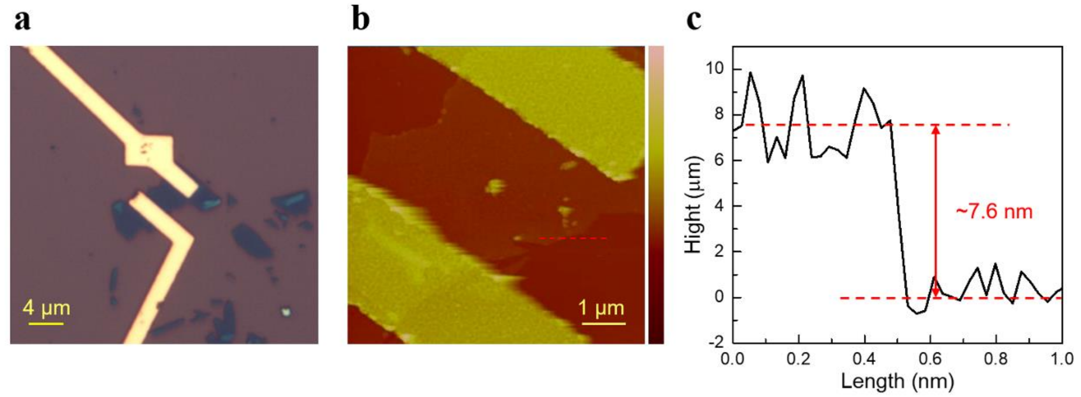

Supplementary Figure 11 Device morphology of device #1. OM (**a**) and AFM (**b**) images of the device. (**c**) Corresponding line profile in (**b**). The channel length L = 2.88 μm and width W = 3.33 μm. Source data are provided as a Source Data file.

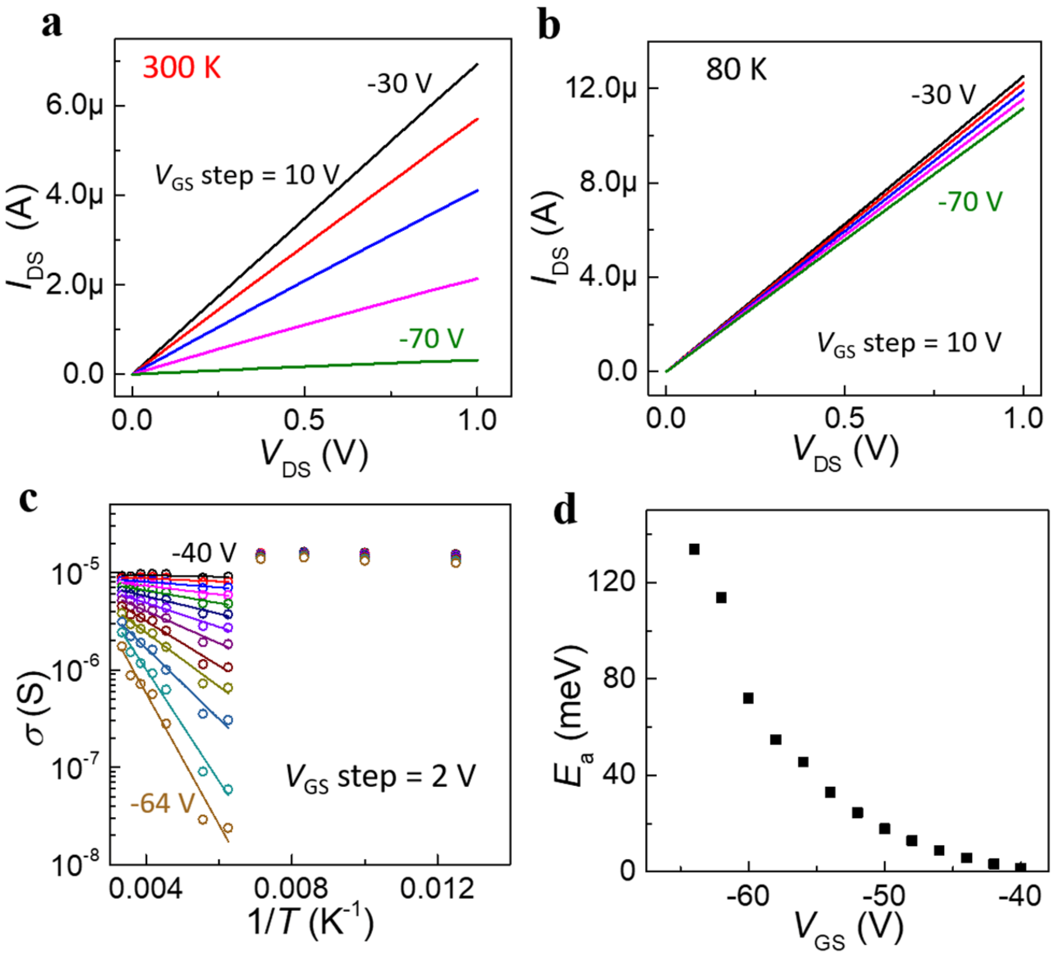

Supplementary Figure 12 Temperature-dependent conductivity of device #2. (**a, b**) I_{DS}-V_{DS} curves of device #2 under various V_{GS} from -30 V to -70 V. T = 300 K and 80 K. (**c**) The temperature and gate voltage dependence of conductivity curves of the device. (**d**) E_a curve as a function of V_{GS}. The two-terminal conductivity is defined as $\sigma = I_{DS}/V_{DS} \times L/W$. The charge transport is thermally activated at 160 K < T < 300 K based on the good agreement of the data with the activation transport model: $G(T) = G_0\exp(-E_a/k_BT)$, where k_B is the Boltzmann constant, E_a is the activation energy, and G_0 is the fitting parameter. The activation energy (E_a), specifying the energy difference between the Fermi level and mobility edge, is extracted and analyzed with the thermally activated transport. Source data are provided as a Source Data file.

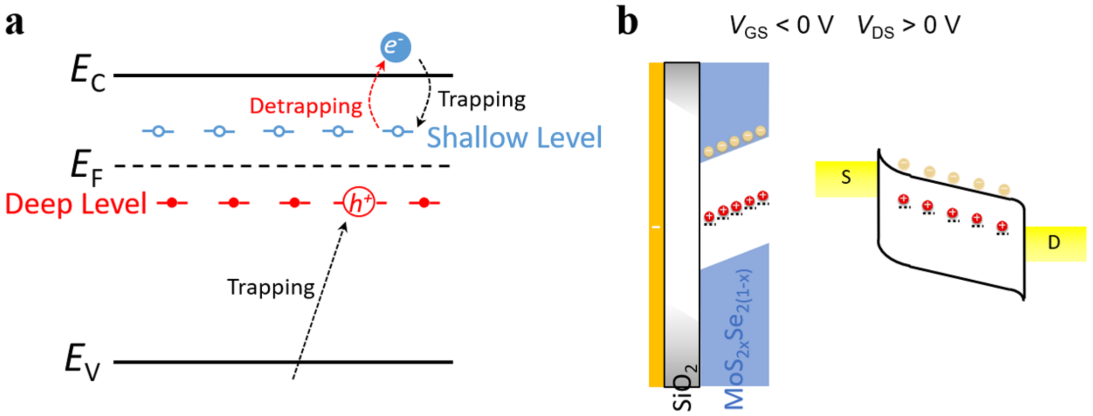

Supplementary Figure 13 Proposed model of hole (minority carrier) trapping and de-trapping for explaining the anomalous electronic behavior. (**a**) Schematic band diagrams of a typical n-type semiconductor with various defect levels. Excess minority carriers (holes) will be captured and stored in deep traps under non-equilibrium conditions. Electrons and holes are represented by blue and red circles with e^- and h^+, respectively. (**b**) Schematic band diagrams of $MoS_{2x}Se_{2(1-x)}$ devices at $V_{GS} < 0$ V, $V_{DS} > 0$ V. Electrons and holes are represented by yellow and red balls, respectively. Black dashed lines in the bandgap denote the empty state (with trapped holes).

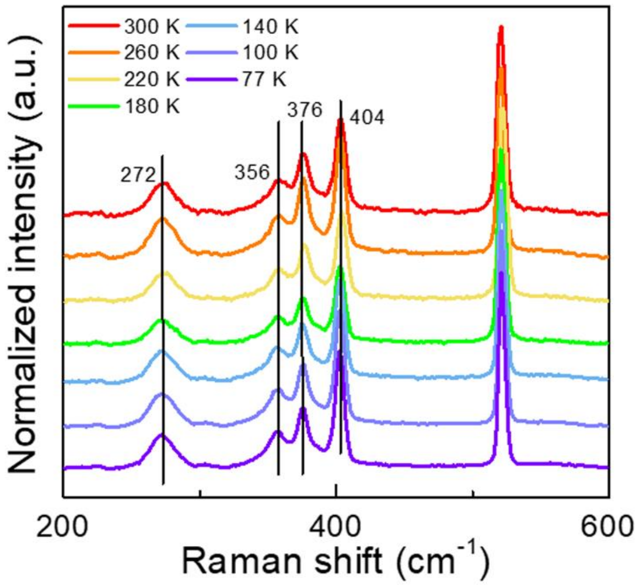

Supplementary Figure 14 Temperature-dependent Raman spectra of $MoS_{2x}Se_{2(1-x)}$ flake. There is neither characteristic peak shift nor new peaks in the process of changing temperature, suggesting no occurrence of structural phase transition. The Raman peaks at 404 cm^{-1}, 376 cm^{-1}, and 272 cm^{-1} are respectively assigned to MoS_2-like A_{1g} mode, MoS_2-like E_{2g} mode, and $MoSe_2$-like A_{1g} mode, which are consistent with the reported $MoS_{2x}Se_{2(1-x)}$ nanosheets[6,7]. Source data are provided as a Source Data file.

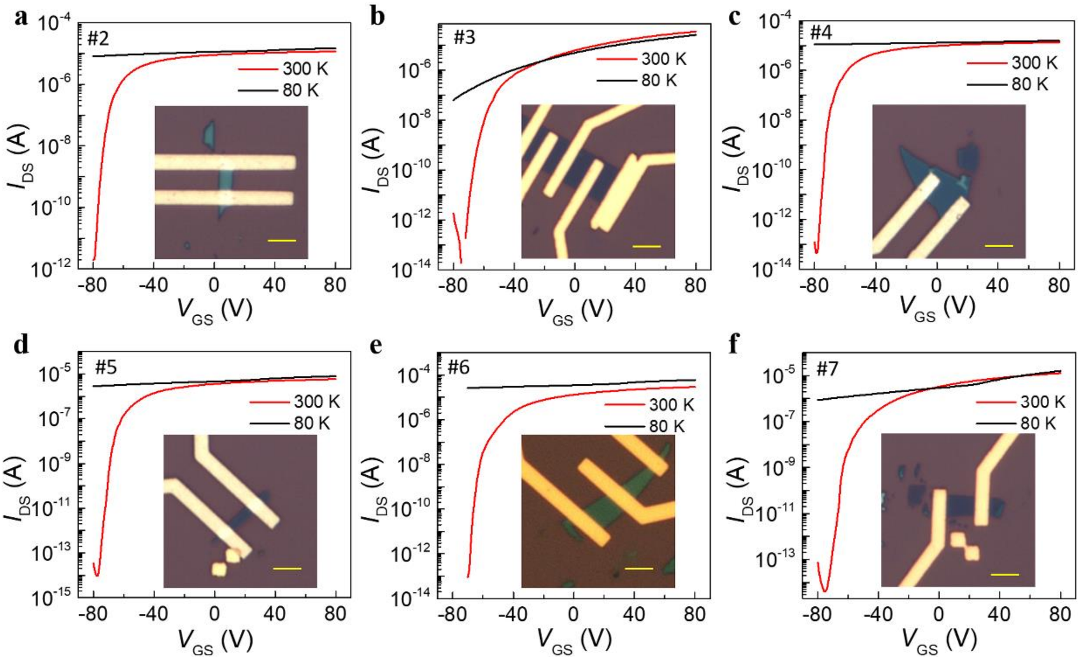

Supplementary Figure 15 I_{DS}-V_{GS} curves of multiple devices at 300 K and 80 K. The OM image of each device is shown in the corresponding inset. Scale bars: 4 μm. The device dimensions: (**a**) L = 2.69 μm, W = 1.63 μm, T = 13.2 nm; (**b**) L = 2.1 μm, W = 5.38 μm, T = 5.2 nm; (**c**) L = 3.44 μm, W = 3.05 μm, T = 8.8 nm; (**d**) L = 3.99 μm, W = 1.39 μm, T = 5.6 nm; (**e**) L = 3.95 μm, W = 3.6 μm, T = 12.9 nm; (**f**) L = 4.12 μm, W = 2.69 μm, T = 5.7 nm. Source data are provided as a Source Data file.

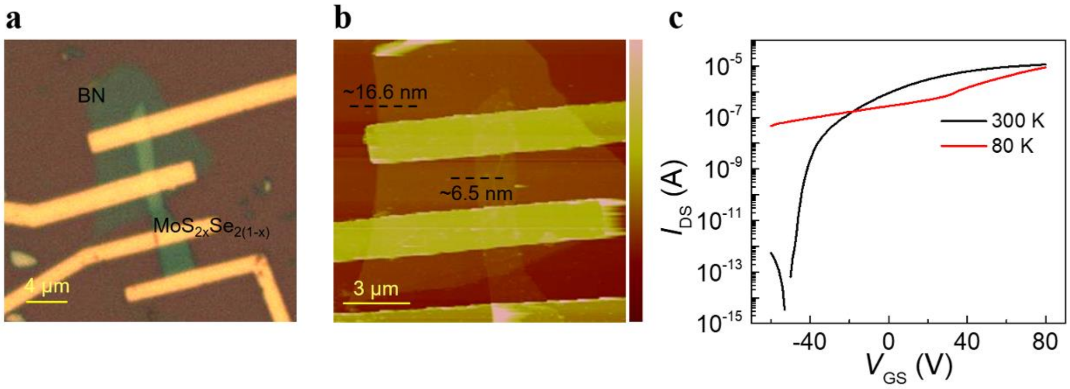

Supplementary Figure 16 Device morphology and electronic transport on BN substrate. OM (**a**) and AFM (**b**) images of the device. (**c**) I_{DS}-V_{GS} curves of the device at 300 K and 80 K. Source data are provided as a Source Data file.

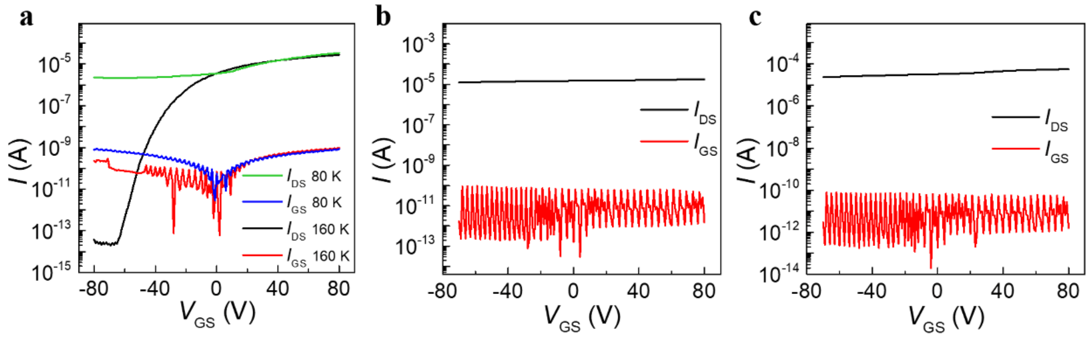

Supplementary Figure 17 The leakage current and source-drain current of three devices (**a-c**) at low temperature. Source data are provided as a Source Data file.

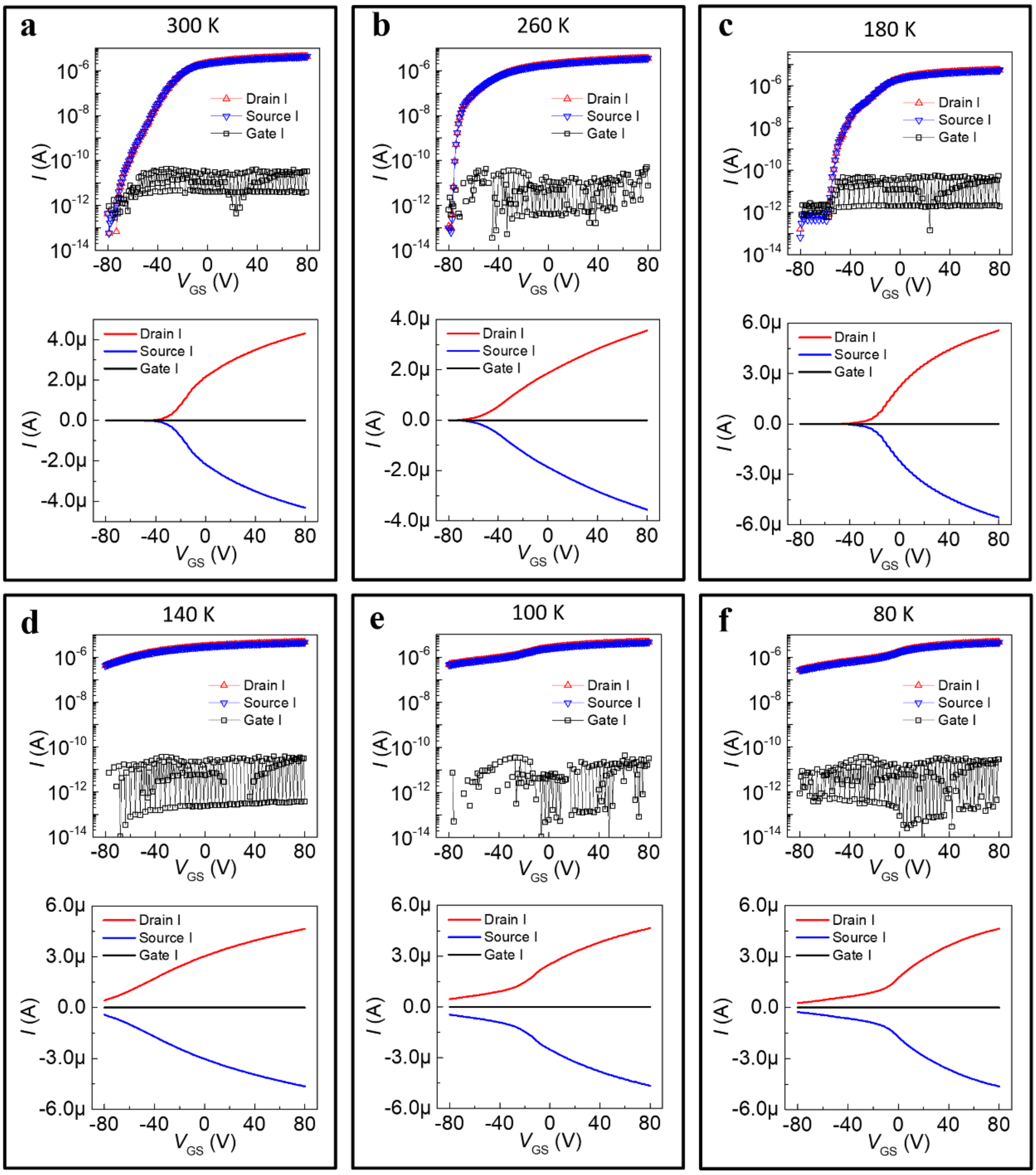

Supplementary Figure 18 All the currents of a typical device at various temperatures (**a**-**f**), plotted in both log (Top) and linear scales (Bottom). The drain current and source current have the same magnitude under various temperatures. Also, the gate current is always much lower than the drain current and source current. These results suggest that there is no leakage current between the gate and drain/source contacts. Source data are provided as a Source Data file.

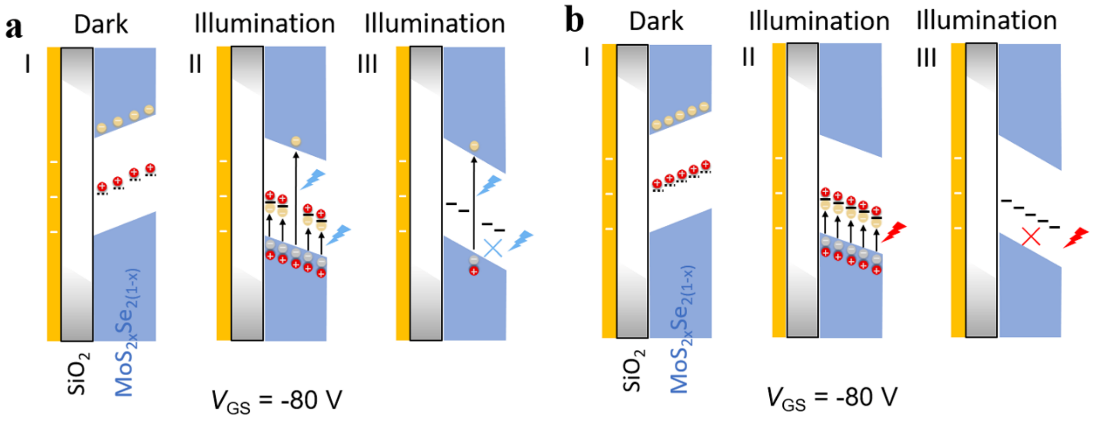

Supplementary Figure 19 Schematic band diagrams of $MoS_{2x}Se_{2(1-x)}$ at V_{GS} = -80 V under dark and illuminated states with 473 nm (**a**) and 1550/1940 nm (**b**) lasers. Progress III represents the subsequent illumination after erasing the trapped holes. Electrons and holes are represented by yellow and red balls. Gray balls are the disappeared electrons or holes. Black dashed and solid lines in the bandgap denote the empty state (with trapped holes) and occupied state (without trapped holes).

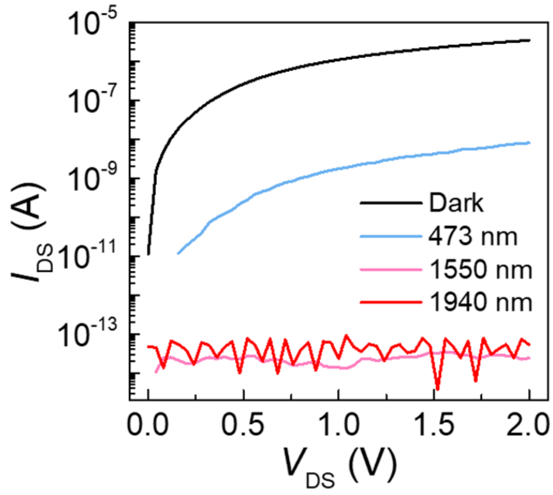

Supplementary Figure 20 Output characteristic curves (I_{DS}-V_{DS}) of device #1 at V_{GS} = -80 V. Source data are provided as a Source Data file.

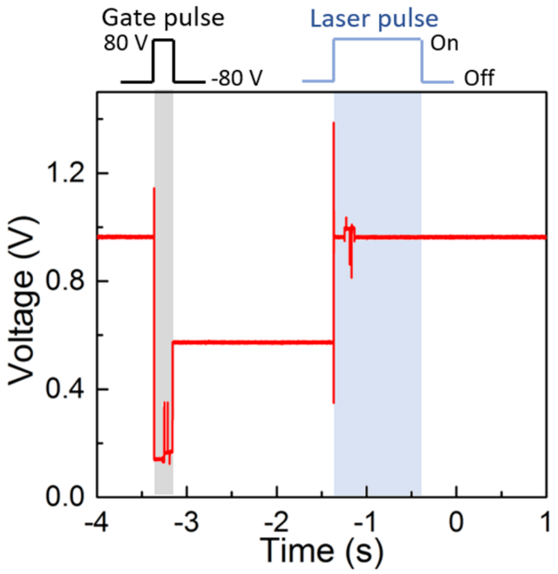

Supplementary Figure 21 A full cycle of response time measurement by an oscilloscope method. The laser wavelength is 1550 nm.

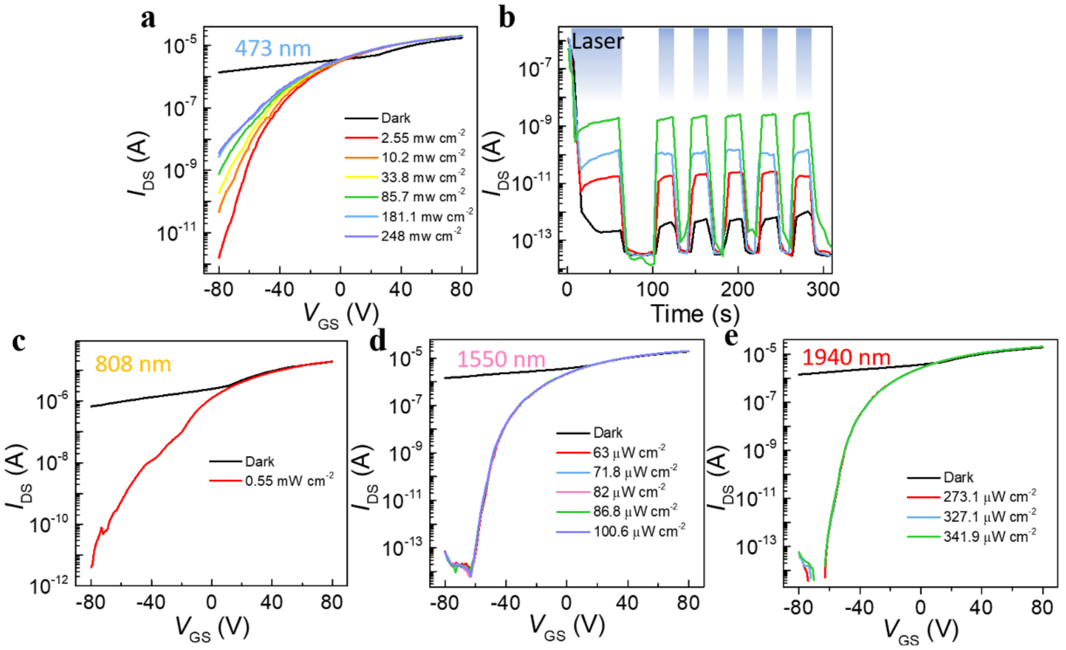

Supplementary Figure 22 Power density-dependent photoresponse of device #1. I_{DS}-V_{GS} curves of the device under dark and illuminated states with 473 nm (**a**), 808 nm (**c**), 1550 nm (**d**), and 1940 nm (**e**) lasers, V_{DS} = 1 V, T = 80 K. (**b**) Time-resolved photoresponse measurement using a 473 nm laser with various power density of 2.55, 10.2, 33.8, and 248 mW cm^{-2} (from green to black). V_{GS} = -80 V. The blue rectangle represents the state of illumination. Source data are provided as a Source Data file.

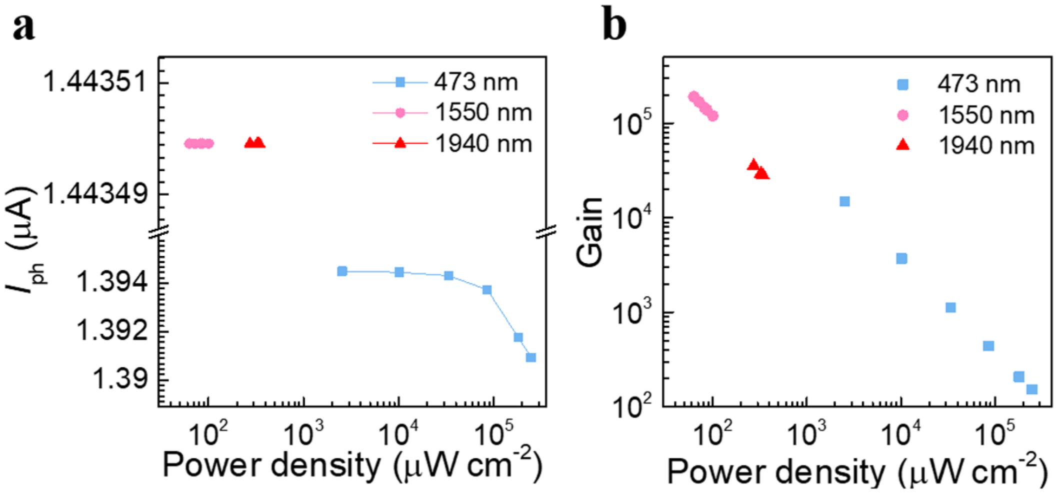

Supplementary Figure 23 Photoresponse performance of device #1. Photocurrent (**a**) and gain (**b**) of the device as a function of power density at V_{GS} = -80 V. Gain is defined as $G = R_{ph}hv/\eta e$[8], where h is the Planck constant, v is the frequency of incident laser, e is the electron charge, and η is the external quantum efficiency which is generally assumed to be 1. Source data are provided as a Source Data file.

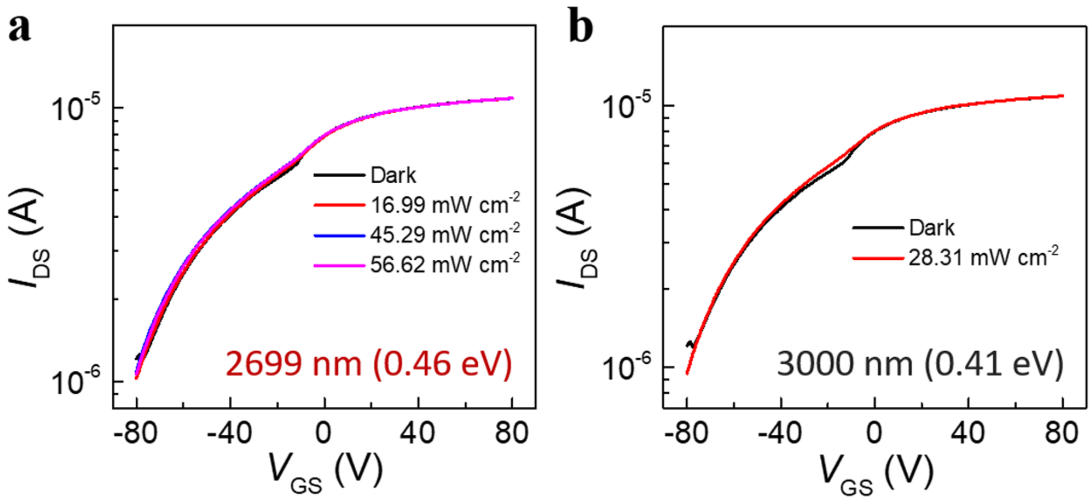

Supplementary Figure 24 I_{DS}-V_{GS} curves of device #1 under dark and illuminated states with 2699 nm (**a**) and 3000 nm (**b**). Source data are provided as a Source Data file.

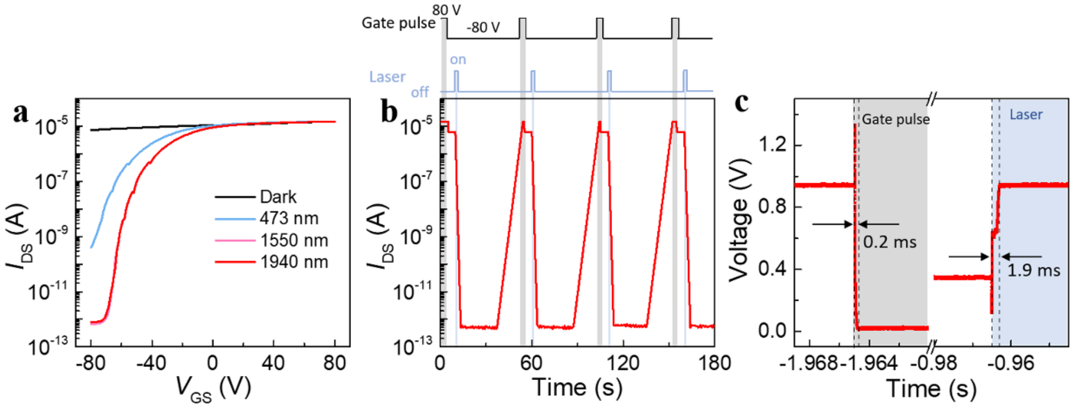

Supplementary Figure 25 Optoelectronic transport of device #2. (**a**) I_{DS}-V_{GS} curves of the device under dark and illuminated states with 473, 1550, and 1940 nm lasers, V_{DS} = 1 V, T = 80 K. (**b**) Switching operation of photodetection using laser pulses (1940 nm, 2s) and gate voltage pulses (V_{GS} = 80 V, 8 s). (**c**) Response time to electro- and photo-excitation. The laser wavelength is 1940 nm. Source data are provided as a Source Data file.

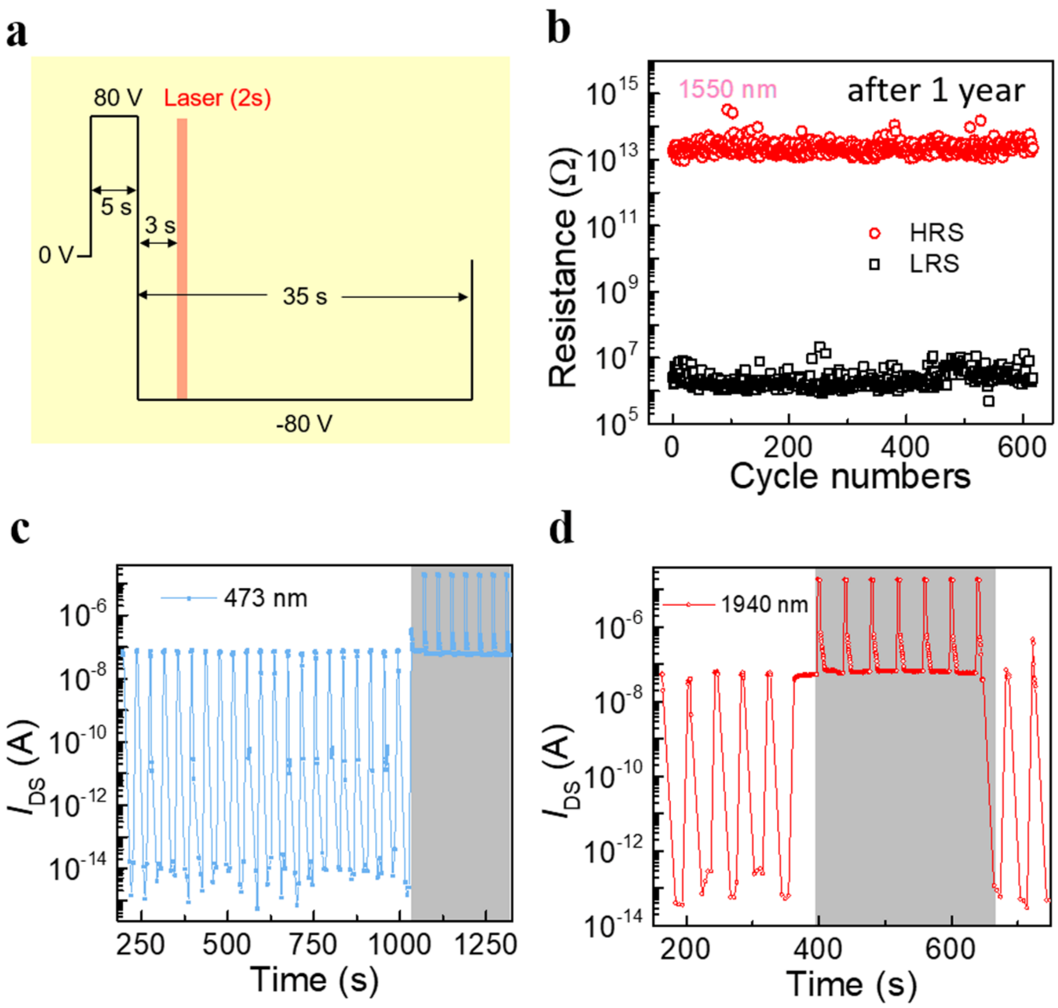

Supplementary Figure 26 Operational endurance of device #1. (**a**) Schematic of one cycle of endurance measurement method. (**b**) Endurance characteristics of the memory for over 600 program/erase cycles, testing again after a year. Laser wavelength and power density are 1550 nm and 100.6 μW cm^{-2}, respectively. (**c, d**) The result of endurance of program/erase operation with 473 nm (**c**) and 1940 nm (**d**) lasers. The power density of lasers is 2.55 mW cm^{-2} and 327.1 μW cm^{-2}, respectively. The shadow region in (**c**) and (**d**) represents the endurance measurement without light (in this case, the trapped holes cannot be erased). Source data are provided as a Source Data file.

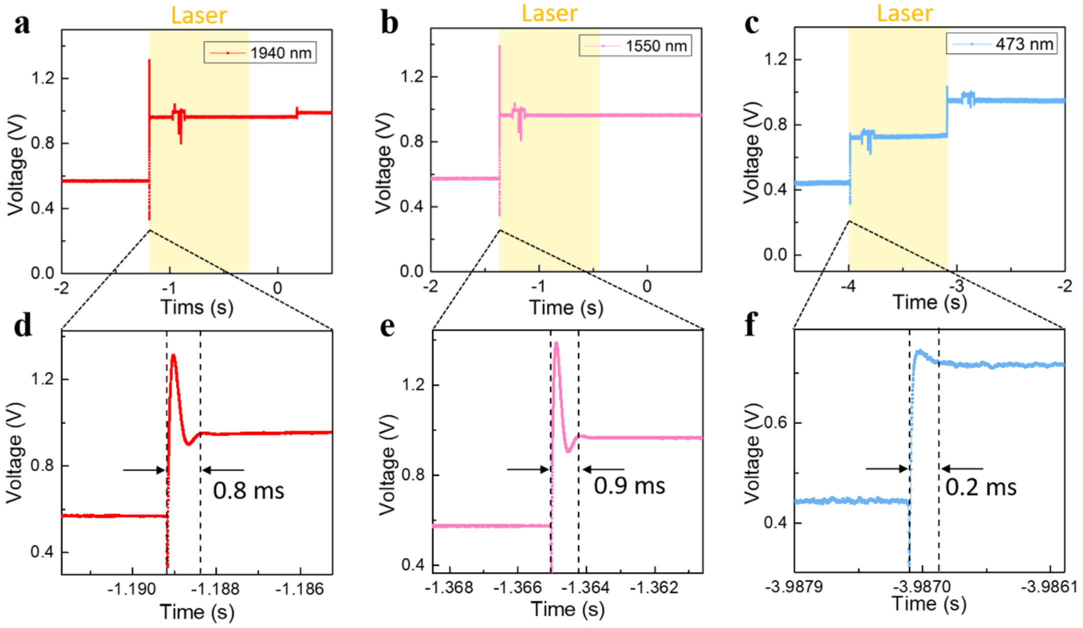

Supplementary Figure 27 Wavelength-dependent erasing speed of device #1. (**a-c**) The erasing speed measurement of the optoelectronic memory with various laser wavelengths (1940, 1550 and 473 nm). (**d-f**) The partial enlarged view of the rising edge region triggered by the laser signal. As for 1550 and 1940 nm laser, the erasing time are almost the same (0.9 and 0.8 ms). This is because the low-energy photons of 1550 nm (0.8 eV) and 1940 nm (0.64 eV) lasers can only pump electrons to the position of in-gap defect levels. Therefore, we consider the erasing time is independent of these infrared lasers. Noteworthily, when the same method is used to identify the erasing time triggered by a 473 nm laser pulse, the obtained time (0.2 ms) is faster than that of infrared illumination conditions. This is because the photoresponse with 473 nm laser is a result of the combined effect of interband and band-edge light absorption.

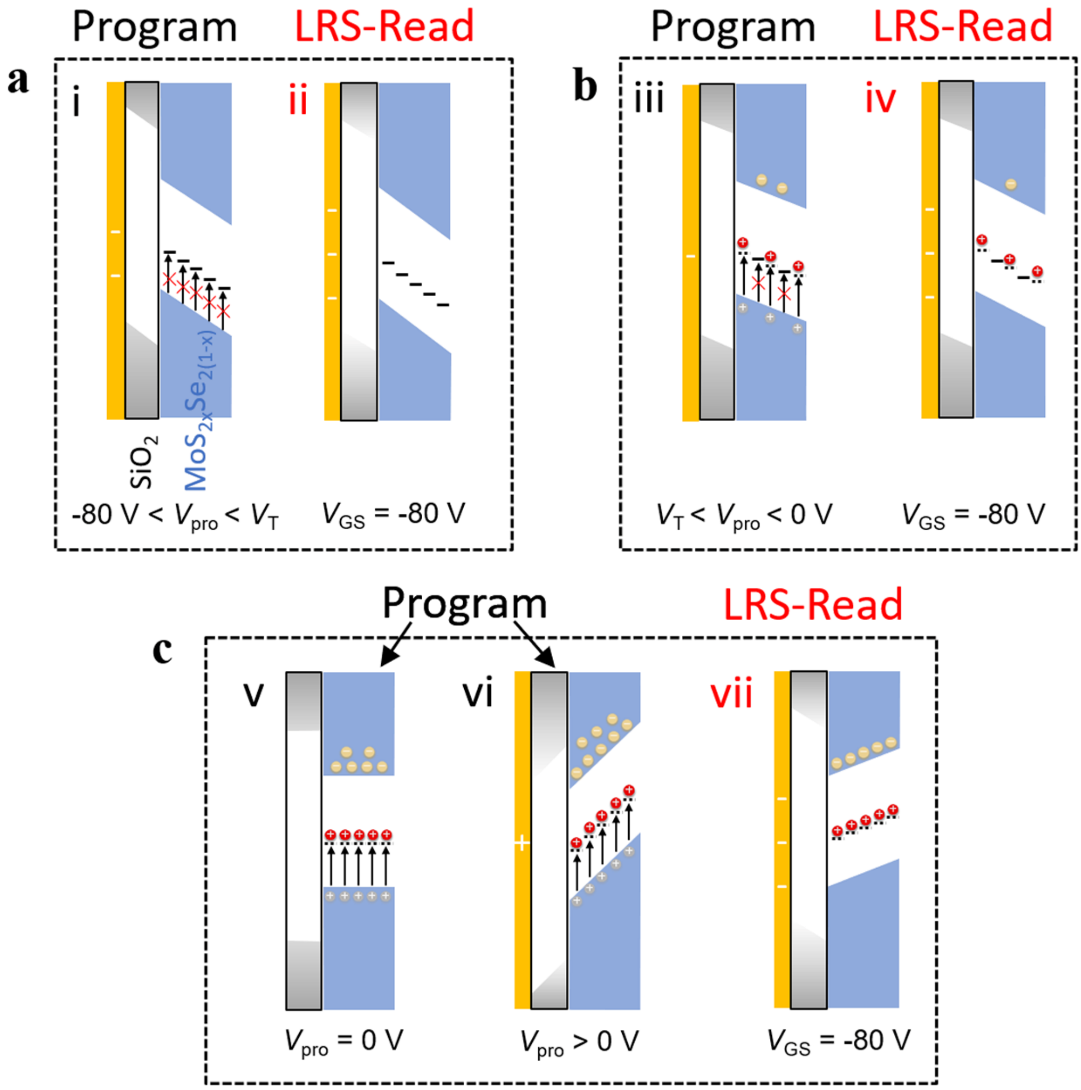

Supplementary Figure 28 Schematic diagrams of gate voltage-dependent memory effect. The schematic diagrams of the programming processes (i, iii, v and vi) under various programming V_{GS} (V_{pro}) and the corresponding LRS-read processes (ii, iv and vii) at V_{GS} = -80 V. (**a**) -80 V < V_{pro} < V_T; (**b**) V_T < V_{pro} < 0 V; (**c**) $V_{pro} \geq 0$ V. V_T is the threshold voltage realizing charge storage. Electrons and holes are represented by yellow and red balls, respectively. Gray balls are the disappeared electrons and holes. Black dashed and solid lines in the bandgap denote the empty and occupied trap states, respectively. Black arrows in the bandgap represent the trapping holes.

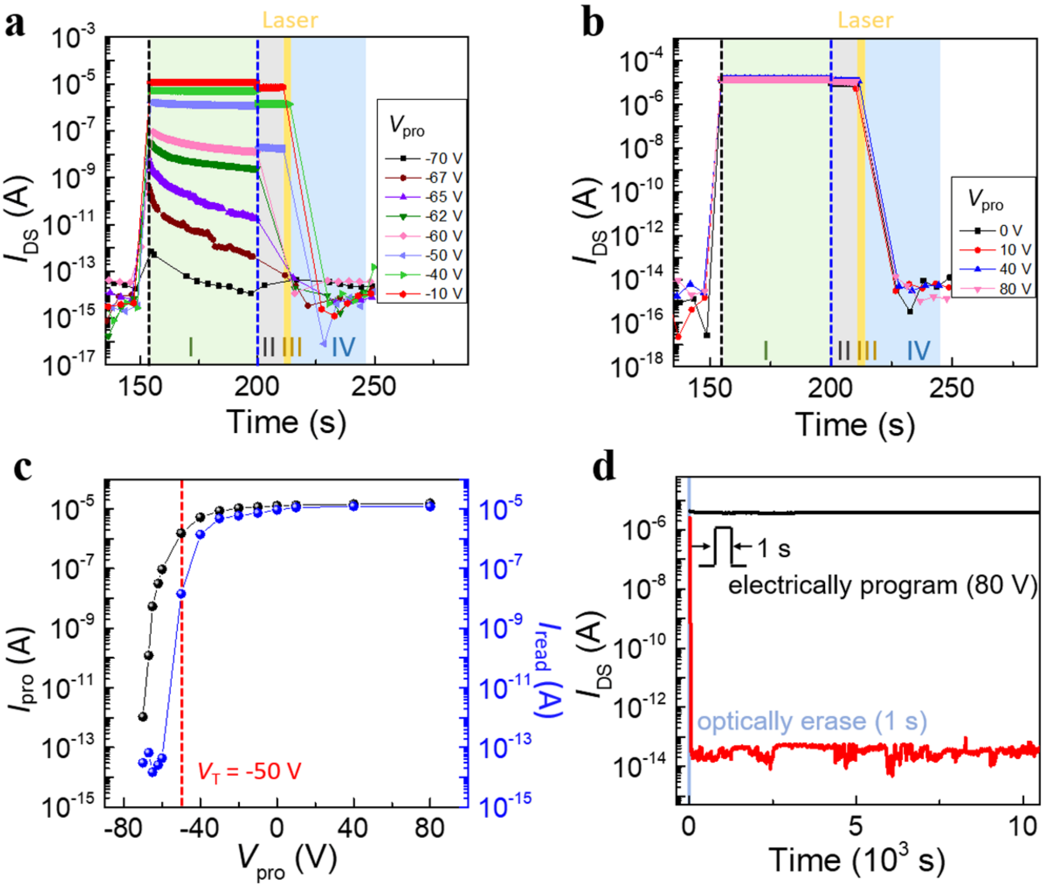

Supplementary Figure 29 Gate voltage-dependent memory effect and performance evaluation of infrared memory of device #2. (**a, b**) Time evolution of current in a single memory cycle with various programming voltages. V_{DS} = 1 V, T = 80 K. Region I, II, III, and IV denote the states of programming, readout (under program state), erasing, and readout (under erase state), respectively. (**c**) Extracted I_{DS} as programming current and read current from (**a**) and (**b**). V_T around -50 V is the threshold voltage realizing charge storage. (**d**) Current retention time of the device after electrical programming and optical erasing with a 1550 nm laser. Source data are provided as a Source Data file.

Supplementary Table 1 Comparison of the figures of merit for different infrared photodetectors based on 2D materials

Materials/structure	Measurement conditions	Photoresponsivity ($A W^{-1}$)	Response time	Ref.
$MoS_{2x}Se_{2(1-x)}$	V_{GS} = -80 V, V_{DS} = 1 V, λ = 1940 nm	5.5×10^4	0.8 ms	This work
	V_{GS} = -80 V, V_{DS} = 1 V, λ = 1550 nm	2.4×10^5	0.9 ms	
Black Phosphorus (BP)	V_{GS} = 3 V, V_{DS} = 0.5 V, λ = 3.39 μm	82		9
Bi_2O_2Se	V_{DS} = 0.6 V, λ = 1200 nm	65	1 ps	10
PbS/MoS_2	V_{GS} = 20 V, V_{DS} = 2 V, λ = 800 nm	4.5×10^4	7.8 ms	11
MoS_2/HgTe quantum dot	V_{GS} = -15 V, V_{DS} = 1 V, λ = 2 μm	5×10^3		12
MoS_2/graphene/WSe_2	V_{GS} = 0 V, V_{DS} = 1 V, λ = 940 nm	0.306		13
h-BN/$MoTe_2$/graphene/ SnS_2/h-BN	V_{GS} = 0 V, V_{DS} = 1 V, λ = 1550 nm	10	3.5 s	14

Supplementary Table 2 Comparison of the figures of merit for different nonvolatile optoelectronic memories

Materials/structure	Program/ erase ratio	Retention time	Programming/ erasing time	Power consumption (programming/erasing)	Response wavelength	Ref.
$MoS_{2x}Se_{2(1-x)}$	10^8	10^4 s	0.3 ms/0.2 ms	3.5 pJ/49 fJ	473 nm	This work
			0.3 ms/0.9 ms	3.5 pJ/8.4 fJ	1550 nm	
			0.3 ms/0.8 ms	3.5 pJ/25 fJ	1940 nm	
$CuIn_7Se_{11}$	<10	50 s			635 nm	15
MoS_2/functionalized substrates	4700	10^4 s			450 nm	16
PbS/MoS_2	600	10^4 s	15.7 ms/0.17 s	420 pJ/1.5 pJ	808 nm<br>1340 nm<br>1550 nm<br>1940 nm	17
WSe_2/BN	10^6	10^4 s	2 s		473 nm<br>515 nm<br>638 nm	18
MoS_2/SWCNTs	10^6	10^3 s	32 ms/0.4 ms		532 nm	19

Supplementary References

1. Kadioglu, Y. et al. Chemical and substitutional doping, and anti-site and vacancy formation in monolayer AlN and GaN. *Phys. Chem. Chem. Phys.* **20**, 16077-16091(2018)
2. Huang, B., Yoon, M., Sumpter, B. G., Wei, S. H. & Liu, F. Alloy engineering of defect properties in semiconductors: suppression of deep levels in transition-metal dichalcogenides. *Phys. Rev. Lett.* **115**, 126806 (2015).
3. Feng, Q. *et al.* Growth of $MoS_{2(1-x)}Se_{2x}$ (x = 0.41-1.00) monolayer alloys with controlled morphology by physical vapor deposition. *ACS Nano* **9**, 7450-7455 (2015).

4. Gong, Y. *et al.* Band gap engineering and layer-by-layer mapping of selenium-doped molybdenum disulfide. *Nano Lett.* **14**, 442-449 (2014).
5. Kim, I. S. *et al.* Influence of stoichiometry on the optical and electrical properties of chemical vapor deposition derived MoS_2. *ACS Nano* **8**, 10551-10558 (2014).
6. Feng, Q. *et al.* Growth of large-area 2D $MoS_{2(1-x)}Se_{2x}$ semiconductor alloys. *Adv. Mater.* **26**, 2648-2653 (2014).
7. Li, H. *et al.* Growth of alloy $MoS_{2x}Se_{2(1-x)}$ nanosheets with fully tunable chemical compositions and optical properties. *J. Am. Chem. Soc.* **136**, 3756-3759 (2014).
8. Zhang, W. *et al.* Role of metal contacts in high-performance phototransistors based on WSe_2 monolayers. *ACS Nano* **8**, 8653-8661 (2014).
9. Guo, Q. et al. Black Phosphorus Mid-Infrared Photodetectors with High Gain. *Nano Lett.* **16**, 4648-4655 (2016).
10. Yin, J. et al. Ultrafast and highly sensitive infrared photodetectors based on two-dimensional oxyselenide crystals. *Nat. Commun.* **9**, 3311 (2018).
11. Wen, Y. et al. Integrated High-Performance Infrared Phototransistor Arrays Composed of Nonlayered PbS-MoS_2 Heterostructures with Edge Contacts. *Nano Lett.* **16**, 6437-6444 (2016).
12. Huo, N., Gupta, S. & Konstantatos, G. MoS_2-HgTe Quantum Dot Hybrid Photodetectors beyond 2 μm. *Adv. Mater.* **29**, 1606576, 2017.
13. Long, M. et al. Broadband Photovoltaic Detectors Based on an Atomically Thin Heterostructure. *Nano Lett.* **16**, 2254-2259 (2016).
14. Li, A. et al. Ultrahigh-Sensitive Broadband Photodetectors Based on Dielectric Shielded $MoTe_2$ /Graphene/SnS_2 p-g-n Junctions. *Adv. Mater.* **31**, 1805656, 2019
15. Lei, S. *et al.* Optoelectronic memory using two-dimensional materials. *Nano Lett.* **15**, 259-265 (2015).
16. Lee, J. *et al.* Monolayer optical memory cells based on artificial trap-mediated charge storage and release. *Nat. Commun.* **8**, 14734 (2017).
17. Wang, Q. *et al.* Nonvolatile infrared memory in MoS_2/PbS van der Waals heterostructures. *Sci. Adv.* **4**, eaap7916 (2018).
18. Xiang, D. *et al.* Two-dimensional multibit optoelectronic memory with broadband spectrum distinction. *Nat. Commun.* **9**, 2966, (2018).
19. Yang, Z. *et al.* High-performance photoinduced memory with ultrafast charge transfer based on MoS_2/SWCNTs network van der Waals heterostructure. *Small* **15**, 1804661 (2019).
